# EGPAware: a European Delphi consensus study on red flags for suspicion of eosinophilic granulomatosis with polyangiitis

**DOI:** 10.1016/j.ero.2025.11.014

**Published:** 2025-12-17

**Authors:** Bernhard Hellmich, Benjamin Terrier, Apostolos Bossios, Christian Domingo, Cristina Ponte, Roser Solans-Laque, Íñigo Rúa-Figueroa, Dimitrios Vassilopoulos, Gabriele Brembilla, Konstantina Kallinikou, Giacomo Emmi, Augusto Vaglio

**Affiliations:** 1 Medius Klinik Kirchheim, Kirchheim unter Teck, Germany; 2 Teaching Hospital of the University of Tübingen, Tübingen, Germany; 3 Cochin Hospital, Paris, France; 4 Karolinska Severe Asthma Centre, Department of Respiratory Medicine and Allergy, Karolinska University Hospital, Stockholm, Sweden; 5 Division for Lung and Airway Research, Institute for Environmental Medicine, Karolinska Institute, Stockholm, Sweden; 6 Lung Laboratory, Centre for Molecular Medicine, Karolinska University Hospital, Stockholm, Sweden; 7 Parc Taulí University Hospital, Parc Taulí Institute of Investigation and Innovation (I3PT-CERCA), Medicine Department, Universitat Autònoma de Barcelona (UAB), Sabadell, Barcelona, Spain; 8 ULS de Santa Maria, Centro Académico de Medicina de Lisboa, Lisbon, Portugal; 9 Faculdade de Medicina, Universidade de Lisboa, Lisbon, Portugal; 10 Vall d’Hebron University Hospital, Barcelona, Spain; 11 Gran Canaria Doctor Negrín Hospital, Las Palmas de Gran Canaria, Spain; 12 National and Kapodistrian University of Athens School of Medicine, Athens, Greece; 13 GlaxoSmithKline, Medical Department, Verona, Italy; 14 GlaxoSmithKline, Respiratory Biologics, Athens, Greece; 15 Department of Medical, Surgical and Health Sciences, University of Trieste, Trieste, Italy; 16 Centre for Inflammatory Diseases, Monash University, Melbourne, VIC, Australia; 17 Department of Medicine, Monash Medical Centre, Melbourne, VIC, Australia; 18 Department of Biomedical, Experimental and Clinical Sciences “Mario Serio”, University of Florence, Florence, Italy; 19 Nephrology and Dialysis Unit, Meyer Children’s Hospital IRCCS, Florence, Italy

## Abstract

**Objectives:**

Diagnosing eosinophilic granulomatosis with polyangiitis (EGPA) is challenging due to its rarity, multisystem involvement, and varied clinical presentation, often resulting in delayed diagnoses and associated complications. EGPAware project aimed to establish a first European Delphi consensus on red flags for raising suspicion of EGPA to assist the prompt identification, referral, and diagnosis of these patients.

**Methods:**

EGPAware was a European Delphi study led by a multidisciplinary committee that developed the survey based on the red flags from a previously published Spanish systematic review and focused on clinical suspicion indicators. Data analysis used descriptive statistics, with consensus defined as ≥70% agreement. Statements with <70% consensus in the first round were revisited again in round 2. In the second round, panellists additionally ranked the 10 most relevant red flags by clinical importance and frequency of observation.

**Results:**

A multidisciplinary European group of 53 physicians with expertise in EGPA completed the 2-round Delphi survey. Consensus was reached on 25 red flags. Mononeuritis multiplex, lung infiltrates/nodules, vasculitis on biopsy, and myeloperoxidase–antineutrophil cytoplasmic antibodies positivity were the highest-ranked signs based on clinical relevance, whereas lung infiltrates/nodules, nasal polyposis, and mononeuritis multiplex were most frequently observed in panellists’ experience.

**Conclusions:**

The EGPAware study provides a useful checklist of consensual red flags to be used in clinical practice to help clinicians identify patients with EGPA early. The study supports a multidisciplinary approach to EGPA diagnosis and management. Further research is recommended to refine diagnostic tools and validate these findings.

**WHAT IS ALREADY KNOWN ON THIS TOPIC**

• The rare nature, numerous clinical manifestations, and their heterogeneity, as well as the limited knowledge among physicians about eosinophilic granulomatosis with polyangiitis (EGPA), often lead to a delayed diagnosis. A previous multidisciplinary consensus study in the Spanish context conducted a systematic literature review to identify and generate a list of red flags for EGPA suspicion.

**WHAT THIS STUDY ADDS**

• This novel study provides an evidence-based and expert-structured consensus-supported list of 25 red flags/signs for suspicion of EGPA agreed upon among European expert clinicians in EGPA.

**HOW THIS STUDY MIGHT AFFECT RESEARCH, PRACTICE OR POLICY**

• The checklist of red flags provided in this consensus is useful for physicians from various specialties, helping them to identify potential EGPA cases earlier in clinical practice.

Alt-text: Unlabelled box dummy alt text

## INTRODUCTION

Eosinophilic granulomatosis with polyangiitis (EGPA), previously referred to as Churg-Strauss syndrome, is a rare autoimmune systemic disease characterised by the presence of blood eosinophilia, eosinophilic tissue infiltration, necrotising vasculitis, and eosinophil-rich granulomatous inflammation [[1], [2], [3]]. It predominantly affects adults between the fifth and seventh decades of life, with a prevalence ranging 1 to 3 cases per million adults worldwide [4]. Its clinical course usually unfolds in 3 stages: allergic or atopic, eosinophilic, and vasculitic [3,5]; with severe asthma being the most common initial clinical feature [[3], [4], [5], [6]].

EGPA negatively impacts patients’ quality of life and requires extensive use of health resources, both of which could be reduced by early diagnosis and timely treatment [7,8]. However, the diagnosis of EGPA is challenging and often delayed due to its rarity, heterogeneous and nonspecific clinical presentation, overlapping clinical and laboratory characteristics with other vasculitides and eosinophilic disorders, and the absence of specific biomarkers [9]. Additionally, the lack of validated diagnostic criteria and referral pathways [9], the lack of, and the often long latency period between asthma and the vasculitis phase (ranging from 3 to 9 years, and up to 30 years) further contribute to delayed diagnosis [10]. Additionally, asthma is the most common initial manifestation of the disease, and the median time from its onset to the diagnosis of EGPA is around 9 [11]. Delayed diagnosis can lead to severe organ damage and complications [1,9], highlighting the importance of improving the early detection of EGPA to facilitate timely, definitive diagnosis and effective treatment [12].

Some studies and consensus have attempted to identify symptoms, signs, and diagnostic tests that enable clinicians to identify potential EGPA cases [1,6,9,13,14]. A Spanish systematic review and expert consensus by Solans-Laqué et al [9] is the most recent publication to produce the most comprehensive list of evidence-based red flags for physicians to use in raising suspicion of EGPA in clinical practice. The EGPAware study considered these red flags for broader validation and refinement in a European setting. The objective of this study was to establish the first European Delphi consensus, through a collaborative multidisciplinary panel of specialists involved in EGPA management, to aid clinicians in identifying red flags for EGPA suspicion in clinical practice.

## METHODS

EGPAware was conducted using the Delphi methodology based on the Accurate Consensus Reporting Document guidelines [15], to ensure consensus-building, which consists of multiple rounds of controlled, anonymous feedback from experts via structured questionnaires. The study was based on a list of red flags gathered from a previous Spanish multidisciplinary project, which was derived from a systematic literature review and local expert consensus [[16], [17], [18]]. There was no formal study protocol established for this project. No patients were involved in the design or conduct of this research.

### Study design

A Scientific Committee (SC) of 10 specialists in EGPA care with backgrounds in respiratory medicine, rheumatology, internal medicine, nephrology, and immunology from Europe (France, Germany, Greece, Italy, Portugal, Spain, and Sweden) was selected based on their recognition within the relevant scientific community (publications, contributions in international and European conferences, input to EGPA clinical practice guidelines development). The SC conceptualised and developed the Delphi survey and established the criteria for selecting panellists: (i) International or national recognition for EGPA expertise; (ii) substantial experience in diagnosing and treating the disease; and (iii) active involvement in research and/or providing recommendations in the field of EGPA.

The initial proposed sample size was 65 panellists (20 rheumatologists; 20 internal medicine specialists; 20 pulmonologists; 5 allergologists) from different countries ensuring a comprehensive representation of Europe and minimising the influence of country-specific practices on the overall results. A total of 60 specialists from the above-mentioned specialties who met the inclusion criteria and had given prior consent to be contacted were invited via email. This process complied with the European General Data Protection Regulation regulations. The email provided details about the project’s objectives and invited them to participate voluntarily and anonymously. Participants’ anonymity was ensured by collecting only the minimum necessary information to track participation between waves and facilitate recontact solely for this project. Participants were informed about the participation clauses, and only those who accepted them were allowed to continue. Responses to the questionnaire were not linked to panellists’ personal information; instead, they were anonymised using an identification number.

Participants in the 1st round were defined as those who accepted the participation clauses and completed 100% of the questionnaire. Because the same participants were expected to complete wave 2, the dropout rate was approximately 11.6%.

Panellists received remuneration if they completed the 2 Delphi rounds as per fair market value. The SC members were remunerated for their work and contribution to the study.

### Delphi survey and data analysis

The Delphi survey was administered in 2 consecutive online rounds (waves [W]): from February to April 2024 (W1) and during August 2024 (W2). The questionnaire was organised into 4 sections (Supplementary Table S1). The first 2 sections were focused on the panellist’s profile and current management of EGPA, respectively. Section 3, focused on the consensus for red flags, was designed based on the list of 40 potential red flags for suspicion from the research by Solans-Laqué et al [9]. The European SC convened to review, enrich, and refine the list of proposed criteria. Red flags were asked considering a patient ≥6 years old with asthma and persistent unexplained blood eosinophil levels of >1000 cells/µL if untreated; or >500 cells/µL if they previously received any medication that may have altered the blood eosinophil count; since the presence of eosinophilia differentiates EGPA from other antineutrophil cytoplasmic antibodies (ANCA)-associated vasculitis, and that asthma is the most common clinical feature at the beginning of the disease.

Section 4 was asked only in wave 2, in which panellists had to rank the list of all red flags, selecting the top 10 most relevant signs for EGPA suspicion based on (i) clinical relevance (understood as their practical importance or applicability to real-world patient care to influence clinical decision-making), and (ii) frequency of observation according to the panellists’ clinical experience.

For statements in sections 2 and 3, a Likert scale ranging from 1 to 9 was used. Results were systematised as follows: 1 to 3: disagreement/not important/useless; 4 to 6: neutral, and 7 to 9: agreement/extremely important/essential. Consensus was established by the SC when ≥70% of the panellists either agreed or disagreed on an item, based on standard practices in Delphi consensus studies. In the scientific literature, consensus thresholds typically range between 65% and 90%, depending on the study design and objectives. The SC selected 70% as an appropriate balance between ensuring a meaningful level of agreement among experts while allowing for some diversity of opinion [19]. Statements that did not reach consensus during W1 were readministered without changes in W2; and additional clarifying notes were added for some statements in W2 (Supplementary Tables S2 and S4). Panellists were invited to suggest additional red flags considered relevant for EGPA suspicion after W1. Those who received SC validation were incorporated into W2.

Data analysis was performed using SPSS version 2.2 (Supplementary Table S1). There were no delays or extensions in the analysis phase.

## RESULTS

### Panellists’ characteristics

Of the 60 invited European panellists with expertise in EGPA, 53 completed the 2 Delphi rounds. Panellists were mainly rheumatologists (35.8%) and pulmonologists (30.2%). They often practiced in the public sector (75.5%), and 92.5% were associated with academic hospitals. Up to 77.3% of the panellists had over 10 years of experience in treating EGPA. In the past 2 years, 50.9% had managed more than 20 patients with EGPA (Table 1).

**Table 1:** Panellist sociodemographic characteristics and EGPA experience

Panellist profile	Results (N = 53)
**Age (y), mean (± SD)**	52.5 (±10.0)
**Country, n (%)**	
Austria	3 (5.7)
Belgium	2 (3.8)
Czech Republic	5 (9.4)
Denmark	3 (5.7)
France	6 (11.3)
Germany	5 (9.4)
Greece	5 (9.4)
Ireland	0 (0.0)
Israel	1 (1.9)
Italy	7 (13.2)
Netherlands	3 (5.7)
Norway	0 (0.0)
Poland	3 (5.7)
Spain	6 (11.3)
Sweden	1 (1.9)
Switzerland	2 (3.8)
United Kingdom	1 (1.9)
**Medical specialty, n (%)**	
Allergologist	3 (5.7)
Immunologist	5 (9.4)
Internal medicine	6 (11.3)
Nephrologist	3 (5.7)
Pulmonologist	16 (30.2)
Rheumatologist	19 (35.8)
Other (Dermatologist)	1 (1.9)
**Experience in EGPA management (y), n (%)**	
<5	1 (1.9)
5-10	11 (20.8)
11-15	14 (26.4)
>15	27 (50.9)
**Patients with EGPA seen, diagnosed, or treated in the last 2 years (number), n (%)**	
<20 patients	26 (49.1)
21-40 patients	15 (28.3)
41-60 patients	4 (7.5)
61-80 patients	2 (3.8)
81-100 patients	2 (3.8)
>100 patients	4 (7.5)

EGPA, eosinophilic granulomatosis with polyangiitis; N, number of panellists; SD, standard deviation; y, years.

**Figure 1:**
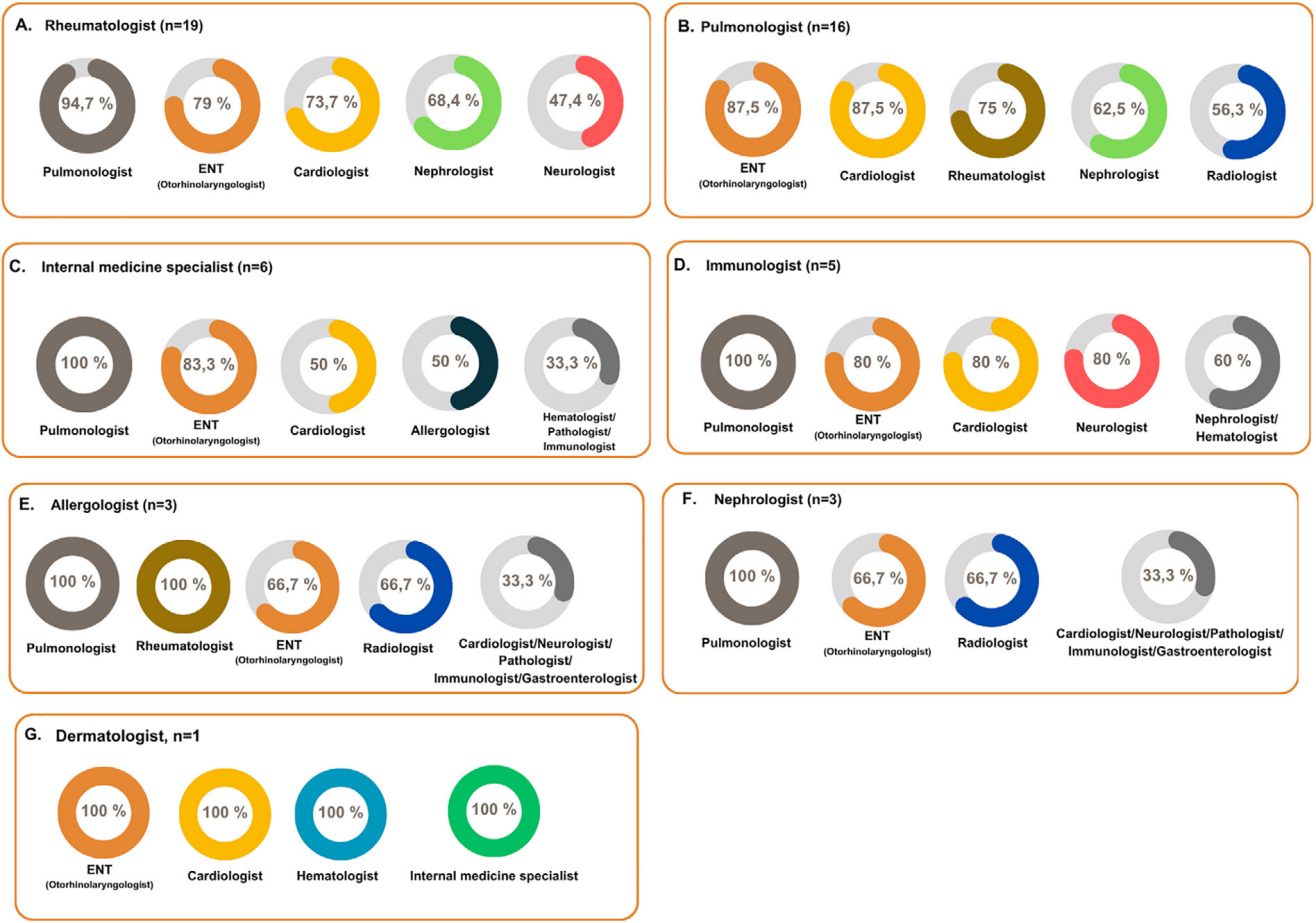
Collaboration with other specialties in the management of EGPA according to each medical specialty from the panel of experts. Note: each participant had to select, of the list, those specialties they collaborate with the most in the management of EGPA. This figure shows the proportion of the specialties with whom each specialty from the panel collaborates the most. Multichoice question. EGPA, eosinophilic granulomatosis with polyangiitis; ENT, ear, nose, and throat specialist; n, number.

All panellists were involved in the diagnosis and follow-up of EGPA, and 98.1% in the treatment of the disease. All of them managed patients with EGPA using a multidisciplinary approach, mainly by consulting colleagues as needed (77.4%). Pulmonology was the most sought-after specialty for collaboration, followed by otorhinolaryngology (ear, nose, and throat [ENT]) and cardiology. Rheumatologists, immunologists, and pulmonologists were the specialists who collaborated with the greatest number of other specialties (Fig 1).

### Perspectives of current management of patients with EGPA from expert’s panel

Panellists agreed that there are no validated diagnostic criteria for EGPA (71.1%). However, classification criteria are often used as diagnostic criteria in common practice (84.9% agreement), even though they were neither designed nor validated for this purpose (eg, the 1990 American College of Rheumatology [ACR] criteria and the 2022 ACR-European Alliance of Associations for Rheumatology [EULAR] criteria) (77.4%). The diagnosis of EGPA becomes complicated due to clinical overlap with other vasculitides or eosinophilic disorders (79.2%) and the lack of uniform clinical criteria and biomarkers to help distinguish EGPA from hypereosinophilic syndrome (83.0%) (Supplementary Table S2).

Potential patients with EGPA should undergo expert evaluation to confirm the diagnosis. However, delays and misdiagnosis are common due to its nonspecific clinical manifestations and low awareness among physicians (88.7%) (Supplementary Table S3).

All panellists considered that the management of EGPA requires an integrated and multidisciplinary approach, although there is a scarcity of EGPA specialists to consult (71.7%). Early diagnosis is associated with improved disease management and a better quality of life for patients (96.2%). A consensus list of criteria for suspecting EGPA could help mitigate the variability in clinicians’ experience and levels of training (94.3%) and facilitate early awareness, reducing referral and diagnosis times for patients with EGPA (92.5%) (Supplementary Table S4).

### Red flags for EGPA suspicion: consensus results and prioritisation

**Figure 2:**
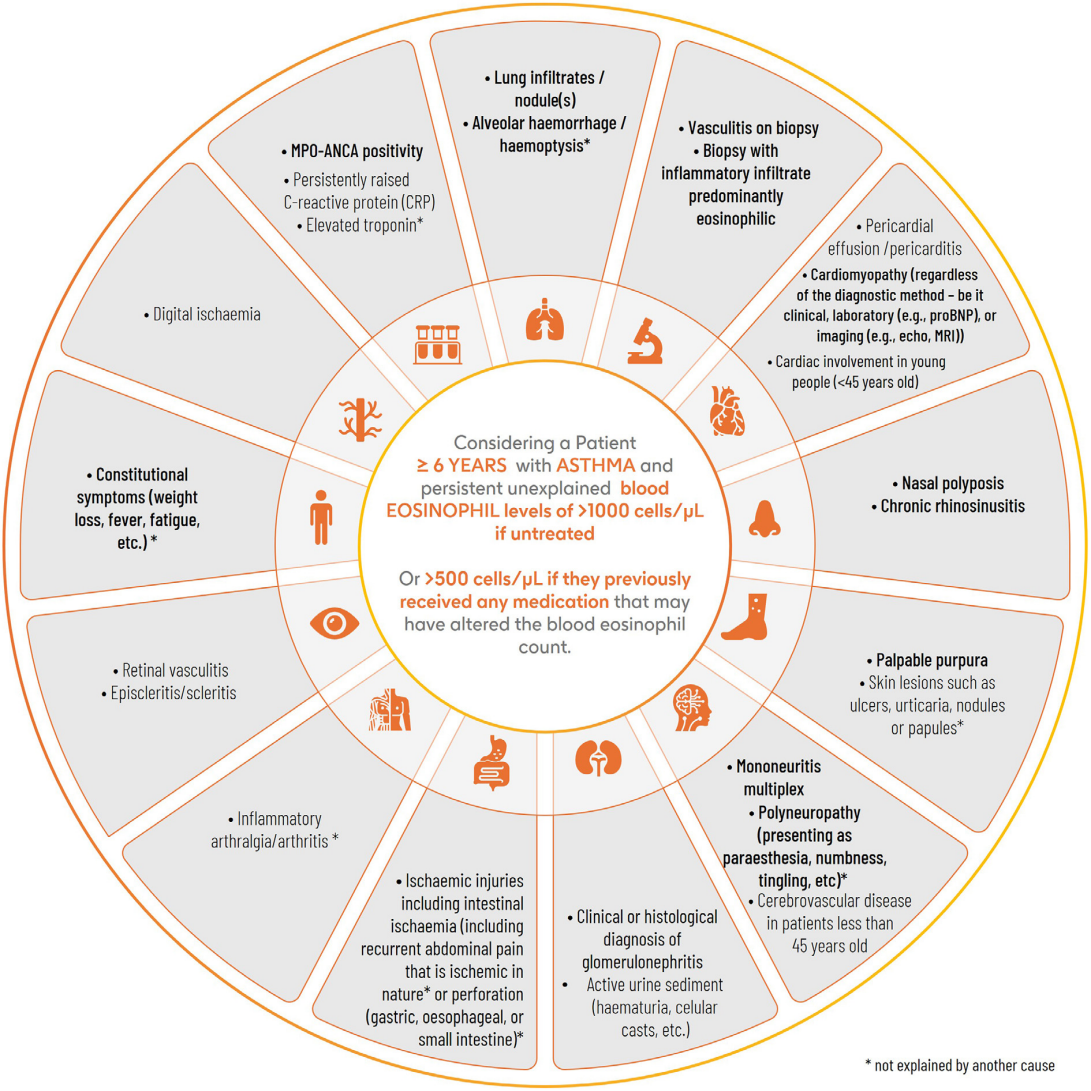
List of agreed red flags for EGPA suspicion. Items in bold correspond to the 12 most relevant red flags based on both clinical relevance and frequency of observation. Clinical relevance is understood as their practical importance or applicability to real-world patient care to influence clinical decision-making, and frequency of observation according to the panellist’s clinical experience. EGPA, eosinophilic granulomatosis with polyangiitis; MPO-ANCA, myeloperoxidase–antineutrophil cytoplasmic antibodies; MRI, magnetic resonance imaging; N, number of panellists; proBNP, B-type natriuretic pro-peptide.

In the context of a patient ≥6 years old with asthma and persistent unexplained blood eosinophil levels of >1000 cells/µL if untreated, or >500 cells/µL if they previously received any medication that may have altered the blood eosinophil count, consensus was reached for 25 of 47 potential red flags for EGPA (Table 2 and Fig 2) (Supplementary Table S5 for detailed information).

**Table 2:** Final consensus^a^ results on agreement regarding red flags for EGPA suspicion

Category	Red flags (on the basis of patient ≥6 y old with asthma and persistent unexplained blood eosinophil levels of >1000 cells/µL if untreated; or >500 cells/µL if they previously received any medication that may have altered the blood eosinophil count)	% of agreement
General	**Constitutional symptoms*****(weight loss, fever, fatigue, etc.)***^b^	79.2
Respiratory	**Lung infiltrates/nodule(s)**	92.5
	**Alveolar haemorrhage/haemoptysis**^b^	79.2
	Wheezing^b^	45.3
	Pleural effusion	45.3
	Chronic cough over 8 wk^b^	32.1
Cardiac	**Cardiomyopathy*****(regardless of the diagnostic method—be it clinical, laboratory [eg, proBNP], or imaging [eg, echo, MRI])***	92.5
	**Cardiac involvement in young people (<45 y old)**^c^	88.7
	**Pericardial effusion/pericarditis**	83.0
	Ischaemic heart disease/arterial occlusion/infarction *(in a patient without identified cardiovascular risk factors*^b^*)*	69.8
	Cardiomegaly^b^	60.4
Vascular	**Digital ischaemia**	73.6
	Venous thrombosis^b^	54.7
Otorhinolaryngological	**Nasal polyposis**	81.1
	**Chronic rhinosinusitis**	71.7
	Chronic media otitis	52.8
Dermatological	**Palpable purpura**	90.6
	**Skin lesions*****(ulcers, urticaria, nodules- or papules***^b^***)***	81.1
Histopathological	**Vasculitis on biopsy**	92.5
	**Biopsy with predominantly eosinophilic inflammatory infiltrate**	90.6
Neurological	**Mononeuritis multiplex**	98.1
	**Polyneuropathy (presenting as paraesthesia, numbness, tingling, etc**^b^**)**	81.1
	**Cerebrovascular disease in patients less than 45 y old**^d^	73.6
	Cerebrovascular disease^b^	60.4
	Chronic paraesthesia^b^	60.4
Renal	**Clinical or histological diagnosis of glomerulonephritis**	89.4
	Renal infarction	47.2
Gastrointestinal	**Ischaemic injuries including intestinal ischaemia*****(including recurrent abdominal pain that is ischaemic in nature, not explained by another cause)*****or perforation*****(gastric, oesophageal, and small intestine***^b^***)***	92.5
	Melena^b^	39.6
	Chronic diarrhoea^b^	18.9
Musculoskeletal	**Inflammatory arthralgia/arthritis^b^**	77.4
	Myositis/myopathy^b^	56.6
Ophthalmological	**Retinal vasculitis**	73.6
	**Episcleritis/scleritis**	71.7
	Inflammatory eye disease *(conjunctivitis, keratitis, episcleritis, scleritis, etc.)*^b^	52.8
	Orbital inflammatory disease/orbital pseudotumor	35.8
	Eye symptoms (red eye, eye pain, decreased vision, etc.)^b^	30.2
Analytical biomarkers	**MPO-ANCA positivity**	92.5
	**Persistently raised C-reactive protein (CRP)**^b^	75.5
	**Elevated troponin**^b^	71.7
	**Active urine sediment*****(haematuria, cellular casts, etc.)***	71.7
	Proteinuria (>500 mg/24 h)	67.9
	Elevated creatinine with or without active urine sediment *(haematuria, proteinuria, cellular casts)*	66.0
	High B-type natriuretic peptide (BNP)^b^	66.0
	Elevated immunoglobulin E^c^	35.8
	PR3-ANCA positivity	11.3
	Rheumatoid factor^b^^,^^c^	9.4

EGPA, eosinophilic granulomatosis with polyangiitis; MPO-ANCA, myeloperoxidase–antineutrophil cytoplasmic antibodies; MRI, magnetic resonance imaging; proBNP, pro B-type natriuretic peptide; PR3-ANCA, proteinase 3 antineutrophil cytoplasmic antibodies; wk, weeks; y, years..

Author-generated table.

a Established when ≥70% of participants agreed (or disagreed) on a statement. Items in bold correspond to those with achieved consensus.

b Not explained by another cause.

c Suggested by the panellists after wave 1 and added after validation from the expert committee.

d Formulated and added in wave 2 by the expert committee based on ‘*Cerebrovascular disease’.*

**Figure 3:**
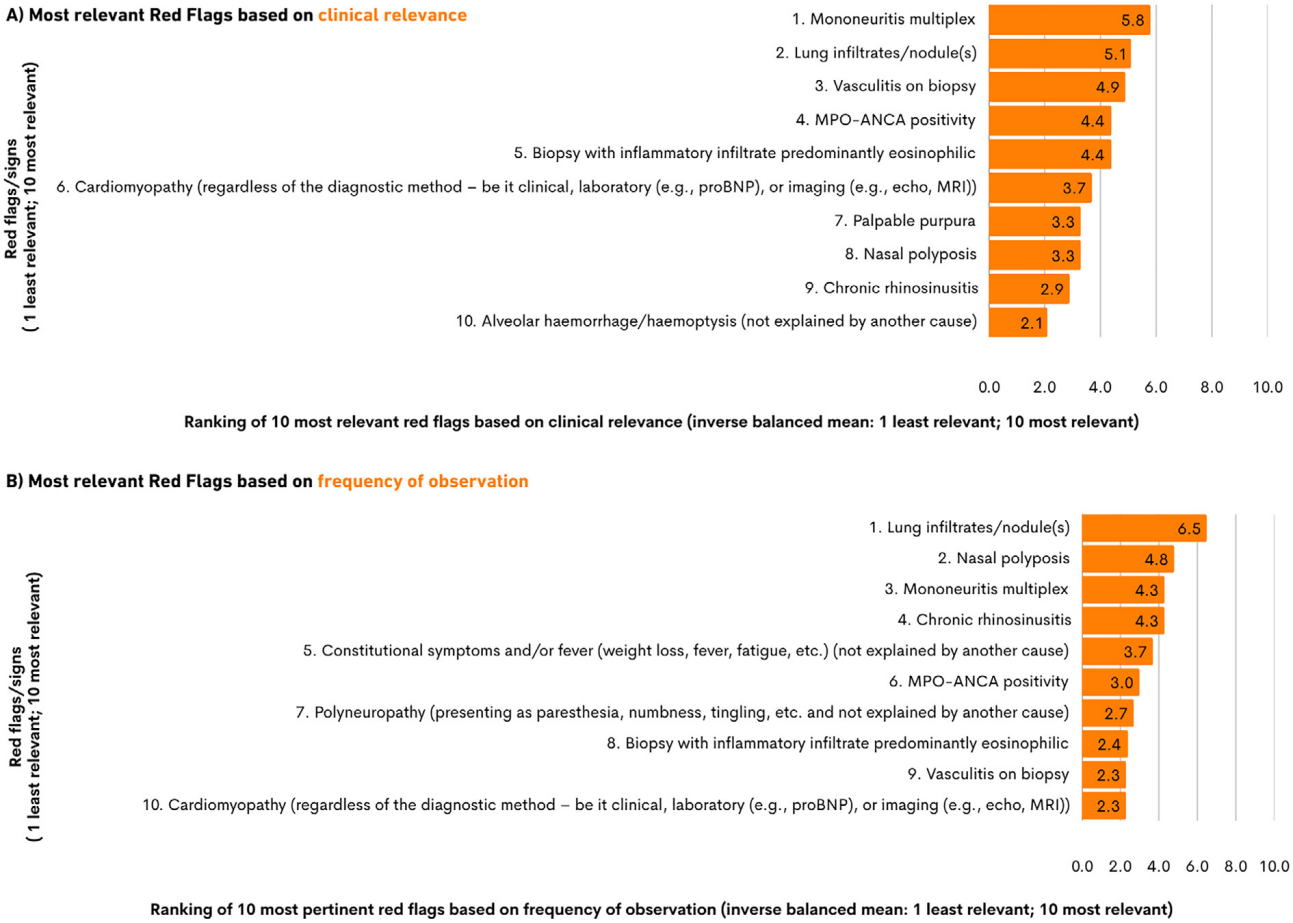
Ten most relevant red flags according to clinical relevance (A) and frequency of observation (B). Notes: ^1^1-least relevant; 10-most relevant; ^2^red flags asked based on a patient ≥6 years old with asthma and persistent unexplained blood eosinophil levels of >1000 cells/µL if untreated; or >500 cells/µL if they previously received any medication that may have altered the blood eosinophil count. ^3^Clinical relevance understood as their practical importance or applicability to real-world patient care to influence clinical decision-making, and frequency of observation according to the panellist’s clinical experience. ^4^ The results shown in the figure were analysed on a total of 52 panellists. MPO-ANCA, myeloperoxidase–antineutrophil cytoplasmic antibodies; MRI, magnetic resonance imaging; proBNP, B-type natriuretic pro-peptide.

The order of priority of the red flags was consistent but varied depending on whether they were ranked by clinical relevance or by frequency of observation (Fig 3). The highest-ranked signs based on clinical relevance included mononeuritis multiplex, lung infiltrate/nodule(s), and vasculitis on biopsy, whereas the most frequently observed signs included lung infiltrate/nodule(s), nasal polyposis, and mononeuritis multiplex.

**Figure 4:**
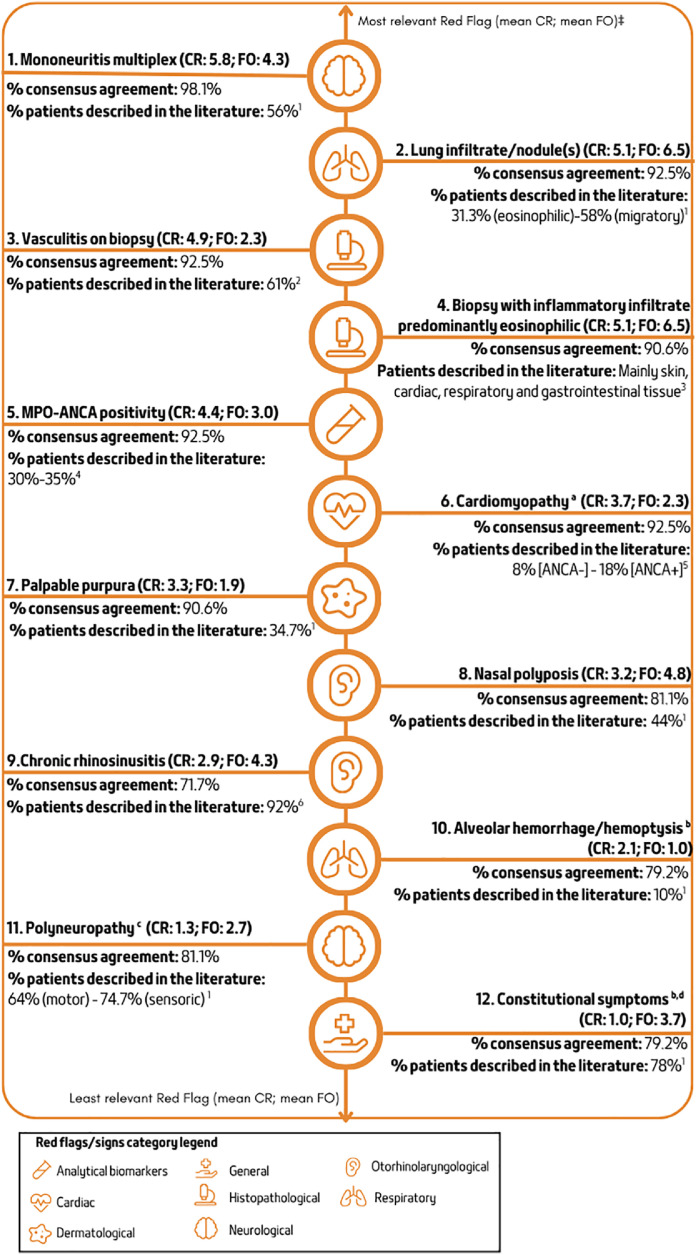
Highest ranked red flags for EGPA suspicion according to clinical relevance (CR) and frequency of observation (FO) (mean)*^†^. Consensus on agreement (% panellists) compared to the proportion of patients (%) with red flag as described in the literature. *Red flags based on a patient ≥6 years old with asthma and persistent unexplained blood eosinophil levels of >1000 cells/µL if untreated; or >500 cells/µL if they previously received any medication that may have altered the blood eosinophil count). ^†^Clinical relevance understood as their practical importance and potential to influence clinical decision-making. ‡To obtain the ranking of the 10 most relevant red flags according to clinical relevance, and to frequency of observation (separately), the 10 most mentioned signs (with the highest percentage of mentions) were used as the starting point. Score of 10 indicated the most relevant sign; whereas 1 indicated less relevant sign. ^a^Regardless of the diagnostic method, be it clinical, laboratory (eg, B-type natriuretic peptide), or imaging (eg, echo, MRI). ^b^Not explained by another cause. ^c^Presenting as paresthesia, numbness, tingling, etc, and not explained by another cause. ^d^Weight loss, fever, fatigue, etc. References: ^1^Moosig et al [20], ^2^Healy et al [21], ^3^Furuta et al [22], ^4^Moiseev et al [23], ^5^Comarmond C et al [11], ^6^Rubenstein et al [24]. ANCA, antineutrophil cytoplasmic antibodies; EGPA, eosinophilic granulomatosis with polyangiitis; MPO-ANCA, myeloperoxidase antineutrophil cytoplasmic antibodies; MRI, magnetic resonance imaging.

The highest-ranked red flags considering both clinical relevance and frequency of observation were, by order: mononeuritis multiplex, lung infiltrates/nodule(s), vasculitis on biopsy, myeloperoxidase (MPO)-ANCA positivity, biopsy with predominantly eosinophilic inflammatory infiltrate, cardiomyopathy, palpable purpura, nasal polyposis, chronic rhinosinusitis, alveolar haemorrhage/haemoptysis, polyneuropathy, and constitutional symptoms (Fig 4) [11,[20], [21], [22], [23], [24]].

## DISCUSSION

To the best of our knowledge, this is the first European study to define a consensus checklist of red flags to aid in EGPA suspicion among physicians in common practice (Fig 2). A multidisciplinary panel of 53 physicians with expertise in EGPA diagnosis and management completed both rounds of the Delphi survey. Challenges in EGPA diagnosis and the lack of awareness among physicians were underscored. Of 47 red flags, 25 achieved consensus, and the highest ranked, based on clinical relevance, included mononeuritis multiplex and lung infiltrate/nodule(s).

The involvement of a broad multidisciplinary group of specialists reflects the real-world multidisciplinary approach to EGPA management, enhancing the study’s distribution applicability. Indeed, pulmonology and rheumatology are the core specialties in EGPA management [7]. Panellists considered that EGPA management requires an integrated multidisciplinary approach, which they already apply in routine practice. This finding aligns with the clinical practice because the initial diagnosis is performed by several specialties. However, based on the authors’ clinical experience, treatment, monitoring, and follow-up are usually done by specialists in secondary or tertiary care, where interdisciplinary discussion is not always required.

Pulmonology and otorhinolaryngology were the most sought-after specialties, probably because ENT manifestations and eosinophilic asthma are among the most common disease manifestations [3,5,6]. Pulmonologists are essential for evaluating pulmonary infiltrates, which can be due to eosinophilic alveolitis, or, less commonly, alveolar haemorrhage or infection. They also optimise inhaled therapies and monitor lung function, whereas otorhinolaryngologists specialists manage chronic rhinosinusitis, nasal polyps, and other ENT-related manifestations [3,5,6,11,25]. Other manifestations, such as mononeuritis multiplex, cardiomyopathy, or skin disease, may also require other specialties intervention. Therefore, in some patients, other specialists are mainly involved at the initial diagnosis stage of EGPA [3,5,6,11,26].

Regarding the red flags, while current classification criteria (ACR/EULAR) are useful for differential diagnosis, these are not intended for diagnosis, and must be applied following the diagnosis of vasculitis. Additionally, EGPA exhibits a heterogeneous clinical presentation. Therefore, a consensus list of red flags could help clinicians suspect of EGPA when a diagnosis of vasculitis has not been made yet. In our study, among neurological signs, polyneuropathy (presenting as paraesthesia, numbness, tingling, etc., with no other identified cause) and mononeuritis multiplex achieved consensus and were included in the ranking of the most relevant signs based on clinical relevance and frequency of observation (Fig 4). Peripheral neuropathy is a common manifestation of EGPA [11,20], and motor and sensoric polyneuropathy affect 64% and 74.7% patients, respectively [20]. Mononeuritis multiplex in its turn has been reported in up to 56% of patients [20], which is consistent with our findings.

Regarding respiratory manifestations, lung involvement beyond asthma occurs in 74.2% of patients [27], with lung infiltrates reported in 31.3% (eosinophilic)—58% (migratory) of them [20]. In our study, lung infiltrates were considered a red flag (92.5% agreement) and included in the prioritisation list as the second most relevant sign. Alveolar haemorrhage/haemoptysis (not explained by another cause) was also included, although it is considered rare in EGPA (10%) [20,28]. Nevertheless, alongside asthma and eosinophilia, this is a strong indicator of EGPA [9].

Regarding histopathological findings, both biopsies with predominantly eosinophilic inflammatory infiltrate and vasculitis on biopsy reached consensus among panellists (>90% agreement) and were included in the list of most relevant signs for suspicion (ranked 3rd and 4th, respectively). This is consistent with the fact that both features play a major role in the disease, and because of their relative specificity [20]. In a large cohort, vasculitis on biopsy was found in 81% of MPO-ANCA+ patients compared to 61% of ANCA− patients [21]. Eosinophilic infiltration varies by tissue and is more common in skin, cardiac, pulmonary, and gastrointestinal involvement, but rare in nerves and kidneys [22].

Of the proposed laboratory biomarkers, MPO-ANCA positivity was included in the overall prioritisation list (ranked 5th). However, it aids in diagnosis and is present in 30%–35% of patients [23].

Concerning cardiac involvement, cardiomyopathy achieved consensus and was ranked 6th in the prioritisation list, which is consistent with the fact that it is the most common cardiac feature [1,29], seen in 19% of ANCA−patients vs 8% of ANCA+ patients [11].

Cutaneous lesions affect between 30% and 50% of the patients [1,27]. In our study, skin lesions (ulcers, urticaria, nodules or papules not explained by another cause) and palpable purpura achieved consensus, with the latter ranked 7th in the prioritisation list. This aligns with the existing literature, which identifies palpable purpura as a key cutaneous manifestation and the most common vasculitis-specific lesion [1,27], affecting 34.7% patients [20].

Of the otolaryngological signs, consensus was reached for nasal polyposis and chronic rhinosinusitis, and these were both voted among the most relevant signs for suspicion. These findings align with the literature because these conditions predominantly affect patients in the early (prodromal) stages of the disease [1], and are present in 44% and 92% of the patients, respectively [20,24]. However, panellists did not reach a consensus on chronic media otitis. This finding could be explained by the fact that this clinical feature appears more often in advanced stages and occurs asynchronously with other systemic features [30].

Lastly, constitutional symptoms also achieved consensus and were included among the most relevant signs, although being nonspecific [12]. According to the literature, general symptoms are present in approximately 78% of patients with EGPA [20], and occur in half of the subjects at relapse [31]. The authors agree that these symptoms should be considered for inclusion in the red flag list for patients with eosinophilic asthma.

Other red flags did not achieve consensus, which may be because they were not deemed specific enough to support their inclusion as red flags (eg, venous thrombosis, chronic diarrhoea not explained by another cause, elevated C-reactive protein and creatinine, with active urine sediment), because these manifestations are not specific to EGPA [12]. However, in a patient with eosinophilia and asthma, these manifestations should also raise suspicion for EGPA [9].

Some factors require consideration when interpreting the findings of this study. For instance, the predominant representation of rheumatologists and pulmonologists as well as the absence of others such as otorhinolaryngologists might influence the generalisability of the findings to other specialties. Additionally, EGPA manifestations vary by stage. For example, asthma and sinusitis are predominant in the prodromal stage, whereas lung infiltration and/or cardiac failure are more common in the eosinophilic phase. Additionally, a positive MPO-ANCA is associated with vasculitis features, whereas an ‘eosinophilic’ phenotype includes other manifestations (eg, cardiomyopathy) [1,29]. However, signs overlap throughout the disease, so the authors believe the prioritisation list remains valid for identifying potential patients with EGPA. Finally, healthcare resources should also be considered, as detection of some signs requires advanced technology (eg, retinal vasculitis), whereas others rely mainly on clinical findings (eg, episcleritis/scleritis) [32].

The EGPAware study has some limitations. First, selection bias may exist since panellists were preselected based on their expertise in EGPA management, which could limit the generalisability of the findings to broader clinical settings. However, this aligns with the Delphi methodology to ensure that consensus is drawn from experts in the study subject [[16], [17], [18]]. Second, the study’s foundation, on previously validated Spanish data, may not fully capture regional differences across Europe in the presentation and management of EGPA. To mitigate this risk, the European members of the SC were invited to review and refine the list during the survey conceptualisation phase; and the panellists were invited to review and suggest additional/omission of red flags in the second round survey. As a result, 4 additional flags were included in the second round of the study after validation by the SC.

The strengths of the EGPAware study include: the use of a well-established Delphi method, which ensured structured consensus-building among a diverse panel of EGPA specialists from several countries. Moreover, EGPAware was developed starting from a previously developed list of red flags, based on evidence, local expert consensus and systematic literature review [9]. Lastly, the participation of a broad European group of experts may enhance applicability.

To the best of the authors’ knowledge, this initiative is the first European study to provide a checklist of red flags developed and agreed upon by a multidisciplinary panel of EGPA-treating physicians to ultimately aid clinicians in identifying early potential patients with EGPA (Fig 2). In fact, to the best of our knowledge, no similar tool has been developed to any systemic rheumatic disease. It highlights several unmet needs and gaps in EGPA diagnosis, particularly the lack of awareness among physicians regarding the disease and the lack of diagnostic criteria. It also underscores the challenges of distinguishing EGPA from other eosinophilic and vasculitic disorders due to overlapping symptoms and the absence of specific biomarkers. The EGPAware study reinforces the need for a multidisciplinary approach to EGPA management, which, together with the provided checklist of red flags, could ultimately improve referral and treatment timelines.

## CRediT authorship contribution statement

**Bernhard Hellmich:** Writing – review & editing, Validation, Supervision, Conceptualization. **Benjamin Terrier:** Writing – review & editing, Validation, Supervision, Conceptualization. **Apostolos Bossios:** Writing – review & editing, Validation, Supervision, Conceptualization. **Christian Domingo:** Writing – review & editing, Validation, Supervision, Conceptualization. **Cristina Ponte:** Writing – review & editing, Validation, Supervision, Conceptualization. **Roser Solans-Laque:** Writing – review & editing, Validation, Supervision, Conceptualization. **Íñigo Rúa-Figueroa:** Writing – review & editing, Validation, Supervision, Conceptualization. **Dimitrios Vassilopoulos:** Writing – review & editing, Validation, Supervision, Conceptualization. **Gabriele Brembilla:** Writing – review & editing, Validation, Supervision, Project administration, Conceptualization. **Konstantina Kallinikou:** Writing – review & editing, Validation, Supervision, Project administration, Conceptualization. **Giacomo Emmi:** Writing – review & editing, Validation, Supervision, Conceptualization. **Augusto Vaglio:** Writing – review & editing, Validation, Supervision, Conceptualization.

## Appendix Supplementary materials

Image, application 1

Image, application 2

## Footnotes

Supplementary material associated with this article can be found in the online version at doi:10.1016/j.ero.2025.11.014.
