# Supplementary material for: EGPAware: a European Delphi consensus study on red flags for suspicion of eosinophilic granulomatosis with polyangiitis
Source: EULAR Rheumatol Open. 2025 Dec 17;2(1):9–18. doi: 10.1016/j.ero.2025.11.014 (PMC13292416; doi:10.1016/j.ero.2025.11.014)
Supplement: Supplementary file 2 [file mmc2.docx]

**EGPAware: a European Delphi consensus study on red flags for suspicion of eosinophilic granulomatosis with polyangiitis (EGPA).**

***Original research article submitted to EULAR Rheumatology Open***

*Supplementary data*

## Table of supplementary material content

[**Supplementary Table S1** 3](#_Toc216687030)

[**Supplementary Table S2** 5](#_Toc216687031)

[**Supplementary Table S3** 6](#_Toc216687032)

[**Supplementary Table S4** 7](#_Toc216687033)

[**Supplementary Table S5** 9](#_Toc216687034)

**Supplementary Table S1. Sections of the EGPAware Delphi survey and data analysis.**

| **Questionnaire section** | **Content and type of questions** | **Data analysis** |
| --- | --- | --- |
| **Section 1** | Panellists’ profile and experience in EGPA management.  Dichotomous (Yes/No) and multiple-choice questions. | Results of dichotomous questions are shown as % of respondents.  Nominal variables were described using numbers and percentage.  Continuous variables were described using mean and standard deviation.  Open-ended responses were analyzed qualitatively to capture additional expert insights, but no formal qualitative analytical methods (e.g., thematic analysis) were applied. |
| **Section 2** | Consensus on current management of patients with EGPA (general management, suspicion and diagnosis).  Likert-scale- type questions with a total of 26 statements. | For statements in sections 2 and 3, a Likert scale ranging from 1 to 9 was used. Results were systematized as follows:   - 1-3: disagreement/not important/useless - 4-6: neutral - 7-9: agreement/extremely important/essential.   Consensus was defined when ≥ 70% of participants agreed (or disagreed) in statement.  Results for consensus statements are shown as % participants.  Open-ended responses were analyzed qualitatively to capture additional expert insights, but no formal qualitative analytical methods (e.g., thematic analysis) were applied. |
| **Section 3** | Consensus on the 47 clinical red flags/signs of suspicion of EGPA (43 asked in Wave 1, and 4 more after Wave 2 added by the panellists and the scientific committee), categorized in 13 areas:   - General - Respiratory - Cardiac - Vascular - Otorhinolaryngological - Dermatological - Histopathological - Neurological - Renal - Gastrointestinal - Musculoskeletal - Ophthalmological - Analytical biomarkers   Likert-scale-type questions.  The red flags were considered based on a patient ≥ 6 years with asthma and persistent unexplained blood eosinophil levels of >1000 cells/µL if untreated. Or >500 cells/µL if they previously received any medication that may have altered the blood eosinophil count. |  |
| **Section 4 (asked only in Wave 2)** | Prioritization of red flags based separately on a) clinical relevance (understood as the practical importance or applicability of these red flags to real-world patient care to influence clinical decision-making) and on b) frequency of observation according to panellists’ practice.    Of the list of 47 red flags, panellists had to select the 10 most relevant indicators for suspected EGPA, and then rank them from 1 (most relevant) to 10 (least relevant). | Results of this section were analysed overall and per specialty.  The 10 most mentioned red flags (with the highest percentage of mentions), for clinical relevance and frequency of observation (separately) were used as the starting point. Subsequently, the mean was calculated for each statement. Next, the inverse balanced mean was calculated to represent the ranking on a scale of 1 (least relevant) to 10 (most relevant).  Results are shown on clinical relevance and frequency of observation separately and considering both criteria. |

**Supplementary Table S2. Wave 2 consensus results on the GENERAL statements about EGPA (Section 2, shown as % panelists)**

| **Statement** | **% disagreement**  **(Totally disagree, 1-3)** | **% neutral (4-6)** | **% agreement (Totally agree, 7-9)** | **Final consensus*** |
| --- | --- | --- | --- | --- |
| Classification criteria are often used as diagnostic criteria. | 3.8% | 11.3% | 84.9% | Agreement |
| There is a lack of uniform diagnostic criteria and biomarkers to help distinguish EGPA from HES (Hypereosinophilic Syndrome). | 5.7% | 11.3% | 83.0% | Agreement |
| EGPA classification criteria are not enough to ensure a timely diagnosis. | 5.7% | 13.2% | 81.1% | Agreement |
| Due to the clinical overlap with other vasculitis or eosinophilic disorders, EGPA diagnosis becomes complicated. | 5.7% | 15.1% | 79.2% | Agreement |
| The classification criteria, such as the 1990 ACR criteria and the 2022 ACR-EULAR criteria, should not be considered diagnostic since they were not originally validated for this purpose. | 1.9% | 20.8% | 77.4% | Agreement |
| There are no validated diagnostic criteria for EGPA. | 18.9% | 9.4% | 71.7% | Agreement |
| In clinical practice, the diagnosis of EGPA is mainly based on clinical characteristics^†^ | 15.1% | 32.1% | 52.8% | Undetermined |
| There is a lack of uniform diagnostic criteria and biomarkers to help distinguish EGPA from the other ANCA-vasculitis. ^†a^ | 24.5% | 22.6% | 52.8% | Undetermined |

Notes: *Consensus was determined when ≥70% participants agreed (or disagreed) in a statement. Otherwise, statements without consensus (<70% agreement or disagreement) are indicated as ‘undetermined’. ^†^Statement that did not reach consensus in the first round and that underwent a second round of voting. ^a^ In the second round, the following explanatory note was included for this item: <<*Please, keep in mind the general spectrum of healthcare professionals, and not your personal experience when answering*.>>

Abbreviations: ACR-EULAR: American College of Rheumatology /European League against Rheumatism; ANCA-vasculitis: Anti-Neutrophil Cytoplasmatic Antibody associated vasculitis; EGPA: Eosinophilic Granulomatosis with Polyangiitis (EGPA).

**Supplementary Table S3. Wave 2 consensus results on the SUSPICION statements about EGPA (Section 2, shown as % panelists)**

| **Statement** | **% disagreement**  **(Totally disagree, 1-3)** | **% neutral (4-6)** | **% agreement (Totally agree, 7-9)** | **Final consensus*** |
| --- | --- | --- | --- | --- |
| Patients with suspected EGPA should undergo expert evaluation to confirm the diagnosis. | 0% | 0% | 100% | Agreement |
| Patients with suspicion of EGPA often experience delays in their diagnosis as further investigation typically occurs only after their symptoms have progressed significantly. | 0% | 11.3% | 88.7% | Agreement |
| A deeper understanding of the disease holds the potential for significant advancements in its early detection. | 3.8% | 7.5% | 88.7% | Agreement |
| A delayed or missed diagnosis in EGPA is common and can be attributed to non-specific clinical manifestations and the limited awareness of this disorder | 5.7% | 5.7% | 88.7% | Agreement |
| In actual clinical practice, the average time between disease onset and its diagnosis may exceed one year. | 3.8% | 9.4% | 86.8% | Agreement |
| At present, EGPA may be underdiagnosed. | 5.7% | 11.3% | 83.0% | Agreement |
| A delay in the diagnosis is common because it often relies on excluding other pathologies. | 5.7% | 13.2% | 81.1% | Agreement |
| The consideration of EGPA is typically ignored until the patient presents with aggravating symptoms. | 5.7% | 22.6% | 71.7% | Agreement |

Notes: *Consensus was determined when ≥70% participants agreed (or disagreed) in a statement. Otherwise, statements without consensus (<70% agreement or disagreement) are indicated as ‘undetermined’.

Abbreviations: EGPA: Eosinophilic Granulomatosis with Polyangiitis (EGPA).

**Supplementary Table S4. Wave 2 consensus results on the DIAGNOSIS statements about EGPA (Section 2, shown as % panelists)**

| **Statement** | **% disagreement**  **(Totally disagree, 1-3)** | **% neutral (4-6)** | **% agreement (Totally agree, 7-9)** | **Final consensus*** |
| --- | --- | --- | --- | --- |
| The management of EGPA requires an integrated and multidisciplinary approach. | 0% | 0% | 100% | Agreement |
| Early diagnosis is linked to better disease management, significantly improving patients' quality of life. | 0% | 3.8% | 96.2% | Agreement |
| A delayed or missed diagnosis is associated with a poorer prognosis and heightened disease damage. | 0% | 3.8% | 96.2% | Agreement |
| A consensus list of suspicion criteria (red flags) for EGPA would help mitigate the variability in experience and training levels among practitioners. | 0% | 5.7% | 94.3% | Agreement |
| A consensus list of suspicion criteria for EGPA would facilitate early awareness, accelerating the referral and diagnosis timelines for patients with EGPA. | 0% | 7.5% | 92.5% | Agreement |
| Timely diagnosis increases the probability of achieving remission in EGPA. | 1.9% | 11.3% | 86.8% | Agreement |
| Specialists occasionally fail to consider EGPA during the diagnostic process, often due to insufficient knowledge about the disease.^a†^ | 5.7% | 17.0% | 77.4% | Agreement |
| The lack of differential diagnosis guidelines and protocols in which EGPA is included ultimately affects diagnostic processes. | 7.5% | 17.0% | 75.5% | Agreement |
| There is a general scarcity of EGPA specialists to consult with. | 5.7% | 22.6% | 71.7% | Agreement |
| Resource limitations in some hospitals can lead to EGPA being inadvertently neglected as a potential diagnosis in favor of more common pathologies.^b†^ | 28.3% | 34.0% | 37.7% | Undetermined |

Notes: *Consensus was determined when ≥70% participants agreed (or disagreed) in a statement. Otherwise, statements without consensus (<70% agreement or disagreement) are indicated as ‘undetermined’. †Statement that did not reach consensus in the first round and that underwent a second round of voting. ^a^In the second round, the following explanatory note was included for this item: <<*When responding, please consider healthcare professionals who may not be specialists or have less experience with EGPA*.>>. ^b^ In the second round, the following explanatory note was included for this item: <<*When responding, please consider resource limitations such as haematologic screening or rapid assess to biopsies>>.*

Abbreviations: EGPA: Eosinophilic Granulomatosis with Polyangiitis (EGPA).

**Supplementary Table S5. Wave 2 consensus results on the RED FLAGS/SIGNS statements for EGPA suspicion (Section 3, shown as % panelists)**

| **Category of Red Flag/sign** | **Red flags/signs** | **% disagreement**  **(Totally disagree, 1-3)** | **% neutral (4-6)** | **% agreement (Totally agree, 7-9)** | **Final consensus*** |
| --- | --- | --- | --- | --- | --- |
| **General** | Constitutional symptoms (weight loss, fever, fatigue, etc.) (not explained by another cause)† | 1.9% | 18.9% | 79.2% | Agreement |
| **Respiratory** | Lung infiltrates/nodule(s) | 0% | 7.5% | 92.5% | Agreement |
|  | Alveolar hemorrhage/hemoptysis (not explained by another cause) | 3.8% | 17.0% | 79.2% | Agreement |
|  | Wheezing (not explained by another cause) † | 9.4% | 45.3% | 45.3% | Undetermined |
|  | Pleural effusion† | 13.2% | 41.5% | 45.3% | Undetermined |
|  | Chronic cough of over 8 weeks of duration (not explained by another cause) † | 13.2% | 54.7% | 32.1% | Undetermined |
| **Cardiac** | Cardiomyopathy (regardless of the diagnostic method - be it clinical, laboratory (e.g., proBNP), or imaging (e.g., echo, MRI)) | 0% | 7.5% | 92.5% | Agreement |
|  | Cardiac involvement in young people (< 45 years old)‡ | 3.8% | 7.5% | 88.7% | Agreement |
|  | Pericardial effusion/pericarditis | 5.7% | 11.3% | 83.0% | Agreement |
|  | Ischemic heart disease/arterial occlusion/infarction (in a patient without identified cardiovascular risk factors and not explained by another cause)† | 7.5% | 11.3% | 69.8% | Undetermined |
|  | Cardiomegaly (not explained by another cause) † | 11.3% | 28.3% | 60.4% | Undetermined |
| **Vascular** | Digital ischemia† | 3.8% | 22.6% | 73.6% | Agreement |
|  | Venous thrombosis (not explained by another cause) † | 3.8% | 41.5% | 54.7% | Undetermined |
| **Otorhinolaryngological** | Nasal polyposis | 3.8% | 15.1% | 81.1% | Agreement |
|  | Chronic rhinosinusitis | 3.8% | 24.5% | 71.7% | Agreement |
|  | Chronic media otitis† | 11.3% | 35.8% | 52.8% | Undetermined |
| **Dermatological** | Palpable purpura | 1.9% | 7.5% | 90.6% | Agreement |
|  | Skin lesions (ulcers, urticaria, nodules, or papules not explained by another cause) | 1.9% | 17.0% | 81.1% | Agreement |
| **Histopathological** | Vasculitis on biopsy | 0% | 7.5% | 92.5% | Agreement |
|  | Biopsy with inflammatory infiltrate predominantly eosinophilic | 3.8% | 5.7% | 90.6% | Agreement |
| **Neurological** | Mononeuritis multiplex | 0% | 1.9% | 98.1% | Agreement |
|  | Polyneuropathy (presenting as paresthesia, numbness, tingling, etc. and not explained by another cause) | 1.9% | 17.0% | 81.1% | Agreement |
|  | Cerebrovascular disease in patients less than 45 years old¥ | 1.9% | 24.5% | 73.6% | Agreement |
|  | Cerebrovascular disease (not explained by any other cause)† | 1.9% | 37.7% | 60.4% | Undetermined |
|  | Chronic paresthesia (not explained by any other cause)† | 9.4% | 30.2% | 60.4% | Undetermined |
| **Renal** | Clinical or histological diagnosis of glomerulonephritis | 3.8% | 11.3% | 84.9% | Agreement |
|  | Renal infarction† | 5.7% | 47.2% | 47.2% | Undetermined |
| **Gastrointestinal** | Ischemic injuries including intestinal ischemia (including recurrent abdominal pain that is ischemic in nature, not explained by another cause) or perforation (gastric, esophageal, and small intestine not explained by another cause) † | 1.9% | 5.7% | 92.5% | Agreement |
|  | Melena (not explained by another cause) † | 15.1% | 45.3% | 39.6% | Undetermined |
|  | Chronic diarrhea (not explained by another cause)† | 30.2% | 50.9% | 18.9% | Undetermined |
| **Musculoskeletal** | Inflammatory arthralgia/arthritis (not explained by another cause) † | 1.9% | 20.8% | 77.4% | Agreement |
|  | Myositis/myopathy (not explained by another cause) † | 1.9% | 41.5% | 56.6% | Undetermined |
| **Ophthalmological** | Retinal vasculitis | 5.7% | 20.8% | 73.6% | Agreement |
|  | Episcleritis/scleritis† | 5.7% | 22.6% | 71.7% | Agreement |
|  | Inflammatory eye disease (conjunctivitis, keratitis, episcleritis, scleritis etc.) (not explained by another cause) † | 9.4% | 37.7% | 52.8% | Undetermined |
|  | Orbital inflammatory disease/orbital pseudotumor† | 20.8% | 43.4% | 35.8% | Undetermined |
|  | Eye symptoms (red eye, eye pain, decreased vision etc.) (not explained by another cause) † | 15.1% | 54.7% | 30.2% | Undetermined |
| **Analytical biomarkers** | MPO-ANCA positivity | 1.9% | 5.7% | 92.5% | Agreement |
|  | Persistently raised C-reactive protein (CRP) (not explained by another cause) † | 7.5% | 17.0% | 75.5% | Agreement |
|  | Elevated troponin (not explained by another cause) † | 5.7% | 22.6% | 71.7% | Agreement |
|  | Active urine sediment (hematuria, cellular casts, etc.) | 3.8% | 24.5% | 71.7% | Agreement |
|  | Proteinuria (>500 mg/24 hours) † | 5.7% | 26.4% | 67.9% | Undetermined |
|  | Elevated creatinine with or without active urine sediment (hematuria, proteinuria, cellular casts) † | 3.8% | 30.2% | 66.0% | Undetermined |
|  | High B-type natriuretic peptide (BNP) (not explained by another cause) † | 5.7% | 28.3% | 66.0% | Undetermined |
|  | Elevated IgE‡ | 22.6% | 41.5% | 35.8% | Undetermined |
|  | PR3-ANCA positivity † | 52.8% | 35.8% | 11.3% | Undetermined |
|  | Rheumatoid factor positivity (not explained by another cause)‡ | 47.2% | 43.4% | 9.4% | Undetermined |

Notes: *Consensus was determined when ≥70% participants agreed (or disagreed) in a statement. Otherwise, statements without consensus (<70% agreement or disagreement) are indicated as ‘undetermined’. ^†^ Statement that did not reach consensus in the first round and that underwent a second round of voting. ^‡^ Red flag/sign added by the panel of experts after the first round (with the previous revision and agreement from the expert committee of the study). ^¥^ Red flag/sign formulated and added in the second round by the expert committee of the study based on the following red flag *<<Cerebrovascular disease (not explained by any other cause)>>.*

Abbreviations: echo: echography; EGPA: Eosinophilic Granulomatosis with Polyangiitis (EGPA); IgE: immunoglobulin type E; MPO-ANCA: Myeloperoxidase Anti-Neutrophil Cytoplasmic Antibodies; MRI: magnetic resonance imaging; PR3-ANCA: proteinase 3 Anti-Neutrophil Cytoplasmic Antibodies; proBNP: pro B-type natriuretic peptide.
